# Carbon quantum dots and chitosan-based heterogeneous silver catalyst for reduction of nitroaromatic compounds

**DOI:** 10.55730/1300-0527.3525

**Published:** 2022-11-18

**Authors:** Farah Samir SALİM, İdris SARGIN, Gülşin ARSLAN

**Affiliations:** Department of Biochemistry, Faculty of Science, Selçuk University, Konya, Turkey

**Keywords:** Chitosan, carbon nanoparticle, *Momordica charantia*, nitro compounds, catalytic reduction

## Abstract

Chitosan plays a crucial role in catalysis, environmental remediation, and sustainable chemistry as a renewable and cationic polysaccharide. Chitosan-based metal catalysts are used in a broad range of chemical transformations. In the study, carbon quantum dots (CQDs) were derived from *Momordica charantia* fruits by microwave irradiation following a green chemistry approach. Three catalysts were designed: Ag(0)-chitosan, Ag(0)-chitosan-*M. charantia* fruit powder, and Ag(0)-chitosan-CQDs. The catalyst supports were prepared by stabilizing CQDs or *M. charantia* powder within the polymeric matrix of chitosan beads. Metallic silver particles were anchored onto glutaraldehyde cross-linked chitosan beads from the aqueous solution of silver nitrate. The heterogeneous silver catalysts were used to reduce toxic nitroaromatics (4-nitrophenol, 2-nitroaniline, 1,2-diamino-4-nitrobenzene, 2,4-dinitrophenol). The regeneration of catalysts was also covered. The reused catalysts retained their catalytic activities after ten cycles. The study suggested that presence of CQDs or *M. charantia* powder could improve the efficiency of the chitosan-based metallic silver catalysts.

## 1. Introduction

Chitosan, as a versatile and functional nontoxic, renewable polysaccharide, exhibits great potential for biomedical applications, pharmaceutical preparation or products and chemical catalysis systems [1]. Many literature reviews have clearly demonstrated its unique position among the natural polymers and its superiority over some synthetic polymers in various fields [2–4].

Chitosan has received much attention in recent years due to its suitability as a functional and renewable polymeric support particularly in heterogeneous catalysis systems [5, 6]. Chitosan, which is a partially-deacetylated and water-soluble form of the parent biopolymer chitin, is obtained easily and inexpensively from hot-alkaline-treated chitin, which is an abundant structural polysaccharide occurring in many organisms including arthropods, molluscs, and fungi. A complete deacetylation of chitin is a rare case and the resulting polymeric structure, which is called chitosan, has randomly distributed β-(1,4)-linked-glucosamine and *N*-acetylglucosamine units [7]. Due to its cationic amino groups, chitosan can dissolve in slightly acidic aqueous medium and form gels. Through electron donating amino and hydroxy groups that it has, chitosan can interact with metal cations, act as a ligand, and form metal complexes, which offers important benefits in preparation of metallic catalysts [8].

Due to its gel-forming properties, chitosan suspensions also function as a dispersive and stabilizing medium for microsized or nanosized particles. Chitosan gel-particle composite suspensions can be moulded into fibres, beads, films, and nanoparticles [9]. Chitosan fibres in the suspensions can be covalently cross-linked through amino groups with dialdehydes, and this gives chitosan chemical and mechanical strength and prevents its dissolution in aqueous media [10, 11].

Because of the properties mentioned above, chitosan is considered an excellent support material for heterogeneous metal catalysis systems. In many studies, chitosan and chitosan derivatives were loaded with metal ions, complexes, and metallic particles and used as catalysts in organic synthesis including carbon-carbon coupling reactions (Suzuki–Miyaura and Heck reactions), oxidations, hydrogenations, and reduction reactions [5].

Metal-catalysed conversion of nitroaromatics into aromatic amines is a useful and significant synthetic route in organic synthesis and environmental remediation of organic pollutants [12]. Nitroaromatic compounds are widely used in various industries and are produced in large quantities and released into the environment mostly through anthropogenic operations [13]. Due to their environmental stability, nonbiodegradability and their detrimental effects on living organisms, the broad use of nitroaromatics poses a serious environmental problem. For example, 4-nitrophenol is carcinogenic and can build up in the food chain, causing various fatal diseases in animals and humans [14]. Reduction of products of nitroaromatics, i.e. aromatic compounds with amino groups, on the other hand, is environmentally safer than the parent compound, and they are useful precursors widely used in synthesis of amides and imines [15].

Homogenous catalysis systems with noble metal particles have been proved efficient in catalytic hydrogenation of nitroaromatics [16]. Despite their higher efficiency, homogenous catalysis systems are not economical because of the difficulties faced during the separation, recovery, and recycling of the used catalyst. Therefore, there has been an increasing demand for heterogeneous catalysis systems due to their easy separation and reusability [17].

Chitosan-based Pd(II), Pd(0), Ru, Pt, Au, and Cu heterogeneous catalysts have been extensively used for reduction of nitroaromatics [17, 18]. Recent studies demonstrated that chitosan-based Ag catalysts, too, which are cheaper than catalysis systems with Pd, Ru, Pt, and Au, can be used effectively in reduction of nitroaromatics [19–23], showing the potential of chitosan-silver heterogeneous catalysis systems. In previous studies, nanosized particles like Fe_3_O_4_, TiO_2_, TiO_2_/ZnO were incorporated into chitosan to enhance the catalytic activity of the chitosan-based metal catalysts [17]. In the past few years, carbon quantum dots (CQDs) have emerged as a new member of fluorescent nanomaterial. Due to their excitation-dependent photoluminescence, high biocompatibility, and low toxicity, CQDs systems are being extensively studied for biosensing, drug delivery, bioimaging, and catalysis [24]. However, a few studies have been conducted on the use of chitosan-CQDs blends as a support material for metal-catalysed organic synthesis [25, 26].

The aim of this study was to test how the coexistence of fine particles, nano- or microsized, would influence the catalytic performance of chitosan-based silver catalysts. In the study, two kinds of particles were incorporated into chitosan support: microsized powder of bitter gourd fruits (*Momordica charantia*) and CQDs derived from powder of bitter gourd fruits (*M. charantia*).

In the study, CQDs were synthesized from dried, mature bitter gourd fruits (*M. charantia*). In the synthesis of CQDs from dried *Momordica charantia* fruits, a facile, straightforward and eco-friendly approach was adopted; CQDs were prepared in water just by microwave irradiation. The conditions under which CQD synthesis was done were optimised by varying the *M. charantia* powder/water ratio, microwave exposure time and the applied microwave energy level. The structural, morphological, fluorescent, and surface chemical properties of the CQDs were identified by fluorescence, UV-vis and FT-IR spectroscopy, TEM images, and the quantum yield calculation. The chitosan-based heterogeneous silver catalysts were prepared by reducing of silver ions onto glutaraldehyde cross-linked chitosan beads (with CQDs or *M. charantia* fruit powder or plain) with a reducing agent (sodium borohydride). These catalysts were identified using various analytical tools (FT-IR, SEM, SEM-EDX) and then were used for reduction of nitroaromatic pollutants (4-nitrophenol, 2-nitroaniline, 1,2-diamino-4-nitrobenzene and 2,4-dinitrophenol) to the corresponding amines in aqueous media in the presence of sodium borohydride. The reusability of the used catalysts was also studied.

## 2. Experimental

### 2.1. Fabrication of carbon quantum dots from *M. Charantia* fruit powder

#### 2.1.1. Preparation of *M. Charantia* fruit powder as carbon precursor

Mature bitter gourd (*Momordica charantia*) fruits were dried at room temperature and then crushed to a fine powder and sieved (with a 100-μm sieve). This sample was also used as carbon precursor for fabrication of CQDs.

#### 2.1.2. Optimization of microwave-assisted fabrication of CQDs from *M. charantia* powder

CQDs from *M. charantia* mature fruits were fabricated in water using a microwave-assisted eco-friendly method. This solvent-free approach involves the exposure of *M. charantia* powder dispersed in water to microwave (MW) irradiation in a microwave oven (CEM Mars 5). The conditions for fabrication of CQDs were first optimized. The optimization experiments were carried to evaluate the combined effect of three variables: microwave irradiation time, solid–liquid ratio (i.e. the mass of *M. charantia* powder–water ratio), and microwave irradiation power (i.e. 400, 800 and 1600 W).

The typical procedure for fabrication of CQDs was as follows:

The typical solution-based strategy for CQD synthesis from *M. charantia* powder was as follows. *M. charantia* powder dispersed in water was placed into a flask and irradiated in a microwave digestion oven. After the microwave exposure, the plant debris was removed by centrifugation (at 3260 × *g*) and the resulting supernatant was collected. The crude supernatant solution was then filtrated using a syringe filter (Millex-GP Syringe Filter Unit, 0.22 μm, Millipore Co., Cork, Ireland). To further purify the solution, the resulting filtrate was dialysed against distilled water. The filtrate was transferred into a dialysis tubing (SnakeSkin^™^ Dialysis Tubing, 3,500 MWCO, Thermo Scientific, the USA) and dialyzed against distilled water overnight. The dialysis medium was refreshed four times. Next, the fluorescence emission of CQDs in water was recorded (excitation, λ_ex_, at 365 nm) on a Perkin Elmer LS 55 instrument (Perkin Elmer, Cambridge, the UK). The optimization experimental condition under which the CQDs that gave the highest fluorescence emission was adopted in the next optimization experiment as an optimal parameter. The CQDs were recovered from the solution using lyophilization and stored in and amber glass in the dark.

The optimization experiments were carried out in a stepwise manner by following the typical reaction procedure for each experiment as follows (from 1 to 4).

1. **Solid–liquid ratio:** The ratio of mass of *M. charantia* powder to water volume was studied at varying masses of *M. charantia* powder: 25, 50, 100, 200, and 400 mg in 10 mL of distilled water. The samples were exposed to microwave irradiation for 30 min at 800 W microwave power energy.
2. **Microwave exposure time:** The effect of microwave irradiation time was studied by applying four different microwave exposure times (i.e. 5, 10, 20, and 30 min). For each experiment, *M. charantia* powder (100 mg) in water (10 mL) was placed in a beaker and irradiated at 800 W power energy.
3. **Microwave irradiation energy:** The influence of microwave irradiation energy was tested at different powers. The carbonization of the precursor with water under microwave of different powers (W) was carried out, applying three different powers (i.e., 400, 800, and 1600 W). For each experiment, *M. charantia* powder (100 mg) in water (10 mL) was placed in a beaker and irradiated for 30 min.
4. **Liquid–solid ratio:** The ratio of water volume to the mass of *M. charantia* powder was studied by keeping the *M. charantia* powder mass constant at 100 mg and varying the volume of the water added from 5 mL to 20 mL. In each experiment, the sample was exposed to microwave irradiation for 30 min at 800 W microwave power energy.

#### 2.1.3. Structural and photophysical characterization of CQDs from *M. charantia*

To visualize the CQDs, transmission electron microscopy was employed (TEM, JEOL JEM-2100, UHR–Ultra High-Resolution instrument). Fourier transform infrared (FT-IR) spectroscopy (Bruker Vertex 70 FT-IR spectrometer, in the range of 4000–500 cm^−1^) was used to examine surface atomic groups of CQDs.

Photoluminescence properties of CQDs were examined by ultraviolet-visible spectroscopy (Shimadzu UV1800 UV–vis absorption spectrophotometer, Shimadzu Corp., Japan) and fluorescence spectroscopy (Perkin Elmer LS 55 instrument Perkin Elmer, Cambridge, the UK).

Fluorescence quantum yield measurement of the CQDs was conducted according to the method reported elsewhere [27]. The fluorescence quantum yield measurement was carried out by comparing the integrated photoluminescence intensities and the absorption measurements (<0.1) of the aqueous solutions of CQDs to the reference standard quinine sulphate (*Φ* = 0.54) in 0.1 M sulphuric acid solutions. The fluorescence quantum yield of CQDs was calculated using the following equation.


(1) $$\Phi_{X}=\Phi_{ST}\;({Grad}_{X}/{Grad}_{ST})\;({\eta^{2}}_{X}/{\eta^{2}}_{ST}),$$

where *Φ_X_* denotes the fluorescence quantum yield of CQDs, *Grad* is the slope of the plot of integrated fluorescence intensity versus the absorbance (*ST*: the standard quinine sulphate, *X*: CQDs), η*_ST_* is the refractive index of the solvent (0.1 M H_2_SO_4_, η=1.33), and η_X_ is the refractive index of the solvent (water, η = 1.33).

### 2.2. Preparation of chitosan beads with *M. charantia* powder and chitosan beads with CDs from *M. charantia* powder

Two types of chitosan beads were prepared as described below: chitosan beads with *M. charantia* powder and chitosan beads with CQDs derived from *M. charantia* powder.

#### 2.2.1. Chitosan beads with *M. charantia* powder

Stock solution of chitosan was prepared by dissolving 6.0 g of chitosan (medium molecular weight, deacetylation degree: 75%–85%, Aldrich) in 300 mL of 2% acetic acid solution and stirred on a magnetic stirrer for 24 h at room temperature. Different quantities of *M. charantia* powder (15, 30, 60, and 120 mg) were dispersed in 30-mL chitosan solutions. A high-speed homogenizer was used to improve the dispersibility of *M. charantia* powder in the aqueous chitosan solutions. The chitosan-*M. charantia* powder dispersion was dropped into a coagulation solution (200 mL of water: 300 mL of methanol: 60 g of NaOH). The coagulated chitosan-*M. charantia* powder droplets that had formed were rested in the medium overnight to ensure a complete coagulation of the beads. The chitosan-*M. charantia* powder beads were then recovered by filtration and washed with distilled water to neutrality. The wet chitosan-*M. charantia* powder beads were placed in cross-linking reaction medium (0.9 mL of glutaraldehyde solution, 25%, in 90 mL of methanol) and treated at 70 °C for 6 h in a fume hood. Finally, cross-linked chitosan-*M. charantia* powder beads were collected by filtration, rinsed with ethanol and then water, and air-dried at room temperature.

#### 2.2.2. Chitosan beads with CQDs from *M. charantia* powder

The same procedure was followed for the preparation of chitosan-CQDs beads. Firstly, different quantities of the CQDs (15, 30, 60, and 120 mg) derived from *M. charantia* powder were dispersed in 30-mL chitosan solutions. The rest of the procedure was the same as the one followed in the preparation of chitosan beads with *M. charantia* powder as described above.

The surface features of the chitosan-*M. charantia* powder beads and the chitosan-CQDs beads were examined by FT-IR and SEM analysis.

### 2.3. Preparation of the chitosan bead-supported silver catalyst

The chitosan-*M. charantia* powder beads and the chitosan-CQDs beads were used as catalyst support material for silver particles. The silver catalysts were fabricated by deposing silver particles from AgNO_3_ aqueous solution onto the chitosan-*M. charantia* powder beads or the chitosan-CQDs beads (Table 1). In each experiment, 0.25 g of the support was placed into aqueous solution of AgNO_3_ (83.3 mg of AgNO_3_ in 100 mL of distilled water) and stirred. Subsequently, the reducing agent (10 mL of 0.05 M NaBH_4_ solution) was added into the AgNO_3_ solutions with chitosan-*M. charantia* powder beads and the chitosan-CQDs beads. The resulting mixture was heated at 100 °C for 6 h. Finally, the beads covered with silver particles were collected by filtration and rinsed with distilled water and air-dried at room temperature. The surface characteristics of the dry beads were determined by FT-IR and SEM analyses. The presence of silver on the surface of the chitosan beads was examined by SEM, EDX, and XRD analyses.

### 2.4. Silver catalysed reduction of nitroaromatics to amino-substituted aromatics

The catalytic performance of the silver-loaded chitosan beads was tested in the reduction reactions of 4-nitrophenol (Sigma-Aldrich), 2-nitroaniline (Sigma-Aldrich), 1,2-diamino-4-nitrobenzene (Acros Organics) and 2,4-dinitrophenol (Merck) in presence of sodium borohydride under mild conditions. In a typical run, 20 mg of catalyst was added into 1.0 mL of aqueous solution of nitroaromatic compound (1.0 × 10^−4^ M, at 22 °C), then the reduction reaction was initiated by adding 10 mg of NaBH_4_. As soon as the yellow colour of the reaction medium disappeared, the colourless solution was immediately pipetted out into a quartz UV–vis cuvette. The reduction reactions of the nitroaromatic compounds to their corresponding amino group carrying aromatic derivatives were monitored on a UV–vis spectrophotometer (Shimadzu UV1800 UV–vis absorption spectrophotometer, Shimadzu Corp., Japan). The reduction reactions were monitored as the disappearance of respective absorbance peaks of the nitroaromatic compounds; 2-nitroaniline: 412 nm, 1,2-diamino-4-nitrobenzene: 404 nm, 4-nitrophenol: the initial 318 nm was shifted to 400 nm after addition of NaBH_4_ because of the formation of intermediate phenolate molecule 4-dinitrophenolate, 2,4-dinitrophenol: the initial 358 nm was shifted to 436 nm after the addition of NaBH_4_ because of the formation of intermediate phenolate molecule 2,4-dinitrophenolate [23].

### 2.5. Regeneration of the silver catalysts

To test the reusability of the used catalysts, the same catalyst was used in ten subsequent runs in the reduction reactions of the nitroaromatics. At the end of each run, the catalyst was recovered by filtration and rinsed with distilled water. The regenerated catalyst was used in the next reduction reaction. In a typical run, 20 mg of catalyst was added into 1.0 mL of aqueous solution of nitroaromatic compound (1.0 × 10^−4^ M, at 22°C), then the reduction reaction was initiated by adding 10 mg of NaBH_4_. The reduction reactions were monitored on a Shimadzu UV1800 UV–vis absorption spectrophotometer.

## 3. Results and discussion

### 3.1. Microwave-assisted fabrication of CQDs from *M. charantia* powder

#### 3.1.1. Optimization of microwave-assisted fabrication of CQDs from *M. charantia* powder

A synthetic “wet chemistry” route was used to construct CQDs. CQDs were successfully prepared by microwave irradiation, using *M. charantia* powder as the carbon source and distilled as the solvent. The microwave-assisted carbonization process generated CQDs which were subsequently released into the medium. CQDs in the medium were separated from the plant debris by centrifugation and microfiltration and further purified by dialysis and then eventually dried by lyophilisation. The optimum operational conditions were recorded as follows; microwave power level: 800 W, microwave exposure time: 30 min, mass of dried *M. charantia* powder: 0.1 g and volume of the solvent (distilled water): 10 mL.

In a previous work by Dong et al. (2021), carbon dots were prepared from fresh fruits of *M. charantia* by using a different technique [28]. In that study, the authors prepared fluorescent carbon dots from *M. charantia* for detection of Pd^2+^ and Fe^3+^ ions. The carbon dots were synthesized from chopped fresh bitter gourd fruits (30 g) by hydrothermal treatment in a 50-mL Teflon-lined autoclave (at 210 °C for 11 h). The solution obtained was centrifuged and the supernatant was filtered through a 0.22-μm syringe filter. Finally, the carbon dots were recovered from the filtrate by drying under vacuum.

This study demonstrated that CQDs, also called carbon dots, could be synthesized from *M. charantia* fruits by a labour-saving microwave-assisted procedure in much shorter time using less energy. Like high temperature in hydrothermal synthesis, in microwave-based synthesis microwave irradiation energy induced the condensation of carbonaceous precursors from *M. charantia* debris and crystallization of the graphitic core.

#### 3.1.2. Structural and photophysical characterization of CQDs from *M. charantia*

**Figure 1:**
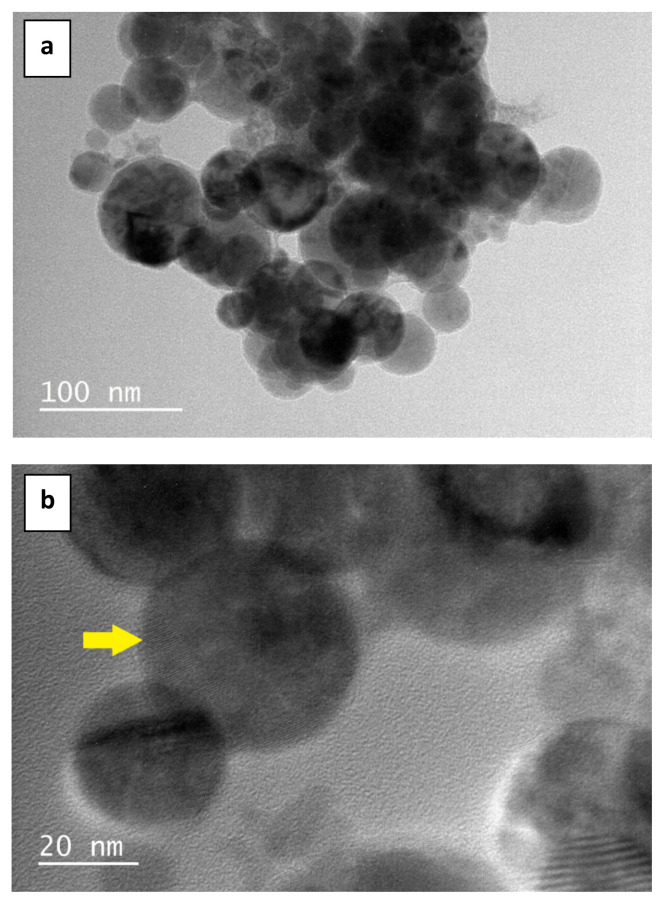
TEM (a) and high-resolution TEM (b) images of CQDs derived from *M. charantia* fruits by microwave irradiation. The arrow points the crystal lattice fringes of the graphitic carbon core (b).

Figures 1a and 1b depict TEM and high-resolution TEM images of CQDs generated from *M. charantia* fruits by microwave irradiation. The images revealed the morphology, size distribution, and crystalline organization of CQDs. CQDs had spherical morphology in varying sizes (Figure 1a). High-resolution TEM image confirmed the periodicity of the graphitic core, reflecting its crystalline nature. The graphite lattice planes of the carbon core are indicated by arrow in Figure 1b.

**Figure 2:**
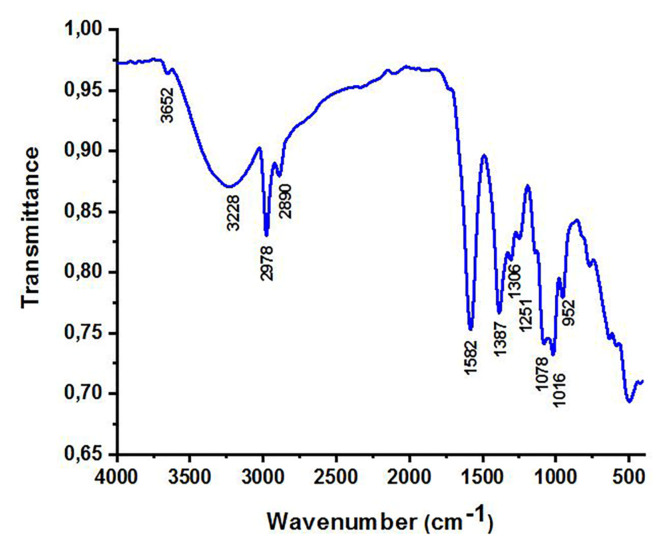
FT-IR spectrum of CQDs derived from *M. charantia* powder.

Fourier-transform infrared spectrum of CQDs derived from *M. charantia* fruits is provided in Figure 2. The spectrum revealed the functional groups corresponding to different atoms present upon the CQDs’ surface. The vibrational bands at 3652 and 3228 cm^−1^ corresponded to O–H stretching vibration and N–H stretching vibration, respectively. The assignment of other peaks in the spectrum are as follows: 2978 cm^−1^ to sp^2^ C–H stretching, 2890 cm^−1^ to sp^3^ C–H stretching, 1582 cm^−1^ to C=C stretching, 1387 cm^−1^ to N–H bending vibrations, 1251 cm^−1^ to C–N stretching, 1078 C–O–C symmetric and asymmetric stretching, and 1016 cm^−1^ to C–OH stretching [28].

**Figure 3:**
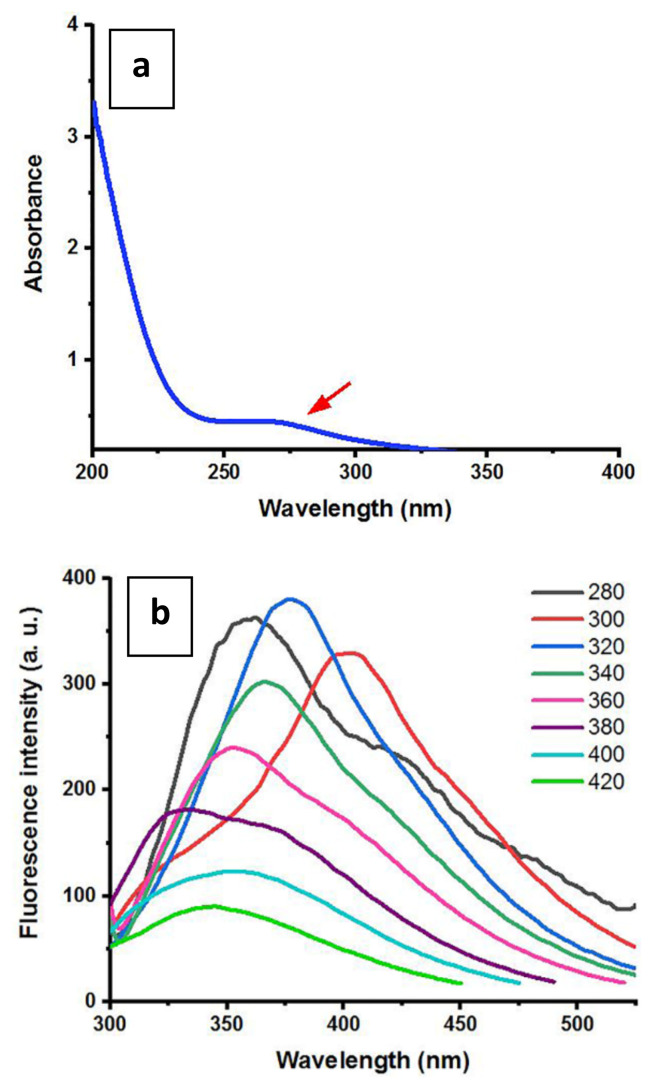
UV-vis absorption spectrum of CQDs derived from *M. charantia* powder. The arrow points the peak that can be ascribed to the n–π* transitions (a). Fluorescence emission spectra of CQDs from *M. charantia* powder at different excitation wavelengths; λex = 280–420 nm. The CQDs showed excitation-dependent photoluminescence (b).

UV–vis absorbance spectrum of the CQDs in Figure 3a depicts a broad absorbance peak that can be ascribed to the presence of multiple energy absorbing groups on the CQDs’ surface. The peak at approximately 270 nm corresponds to the n–π* transitions of carbonyl groups. The absorbance of the CQDs solution decreased as the wavelengths increased and became weaker around 400 nm. The fluorescence emission spectra of the CQDs solutions were recorded at different excitation wavelengths from 280 nm to 420 nm (Figure 3b). Despite their weak absorbance at 400 nm (Figure 3a), the CQDs solutions still had fluorescence emissions as depicted in Figure 3b. The CQDs showed excitation-dependent photoluminescence, which is typical of CQDs. The photoluminescence of CQDs is ascribed to different surface energy states resulting from surface functional moieties and surface defects [28, 29].

Fluorescence quantum yield (*Φ*) of the CQDs was calculated with reference to quinine sulphate solution (*Φ* = 0.54). The quantum yield of the CQDs from dried fruits of *M. charantia* was calculated as 40.38%. Dong et al. (2021) prepared fluorescent carbon dots from fresh fruits of *M. charantia* by using a different technique: hydrothermal treatment [28]. In that study, the authors measured the quantum yield of carbon dots as 11.34% using quinine sulphate as a reference. This demonstrated that the microwave-assisted fabrication method followed in this study could be more convenient for preparation of CQDs from *M. charantia* plant with higher fluorescence emission intensity.

### 3.2. Preparation of the chitosan beads with *M. charantia* powder and the chitosan beads with CQDs from *M. charantia powder*

**Figure 4:**
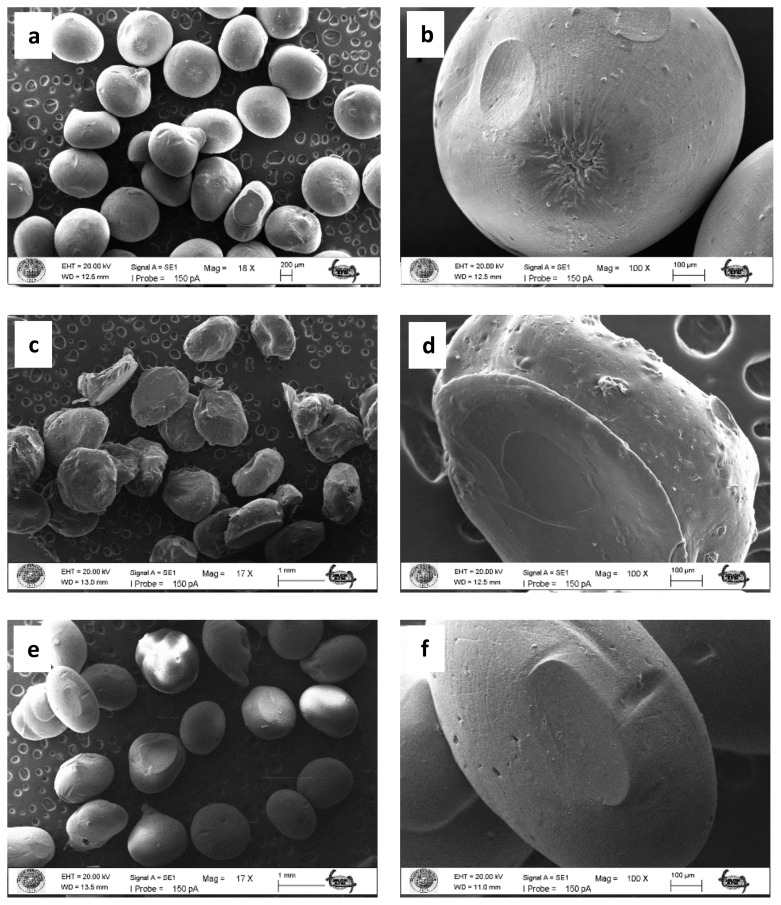
SEM images of the chitosan beads: the plain chitosan beads (without *M. charantia* powder or CQDs) (a, b), the chitosan beads with *M. charantia* powder (c, d), and the chitosan beads with CQDs from *M. charantia* powder (e, f).

The SEM images of the plain chitosan beads, the chitosan beads with *M. charantia* powder and the chitosan beads with CQDs from *M. charantia* powder are depicted in Figures 4a–4f. The plain beads and the beads with CQDs had smooth surface. The surface of the beads with *M. charantia* powder was rough because of the incorporated microsized plant debris.

### 3.3. Preparation of the chitosan bead-supported silver catalysts

**Figure 5:**
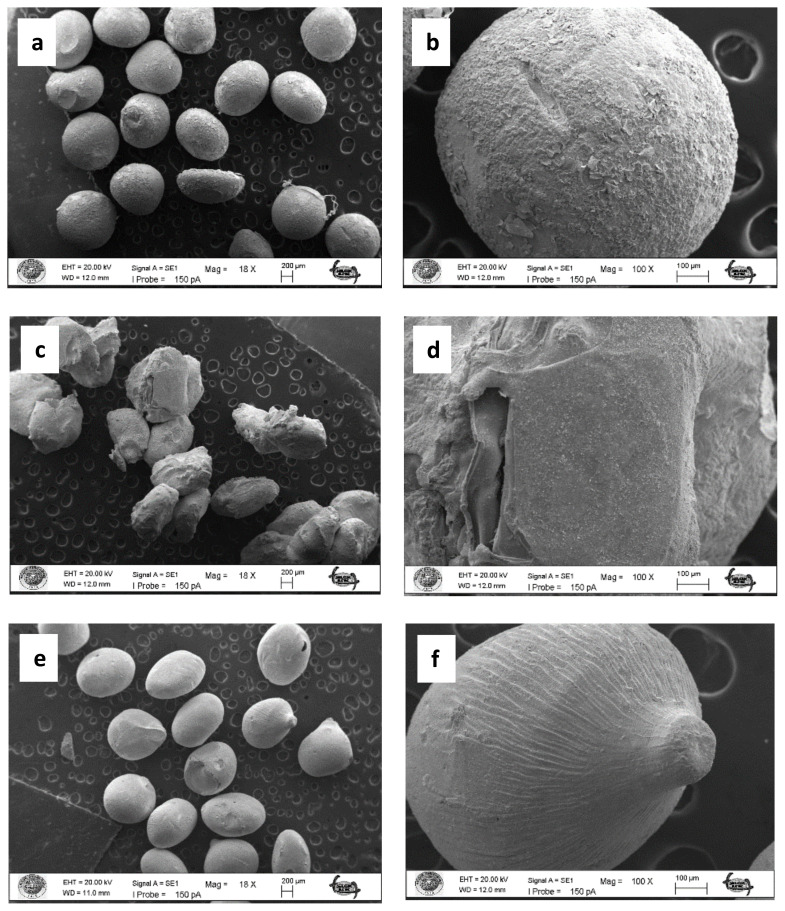
SEM images of the chitosan-based silver catalysts: Ag(0)-chitosan beads (without *M. charantia* powder or CQDs) (a, b), Ag(0)-chitosan-*M. charantia* fruit powder (c, d), and Ag(0)-chitosan-CQDs (e, f).

**Figure 6:**
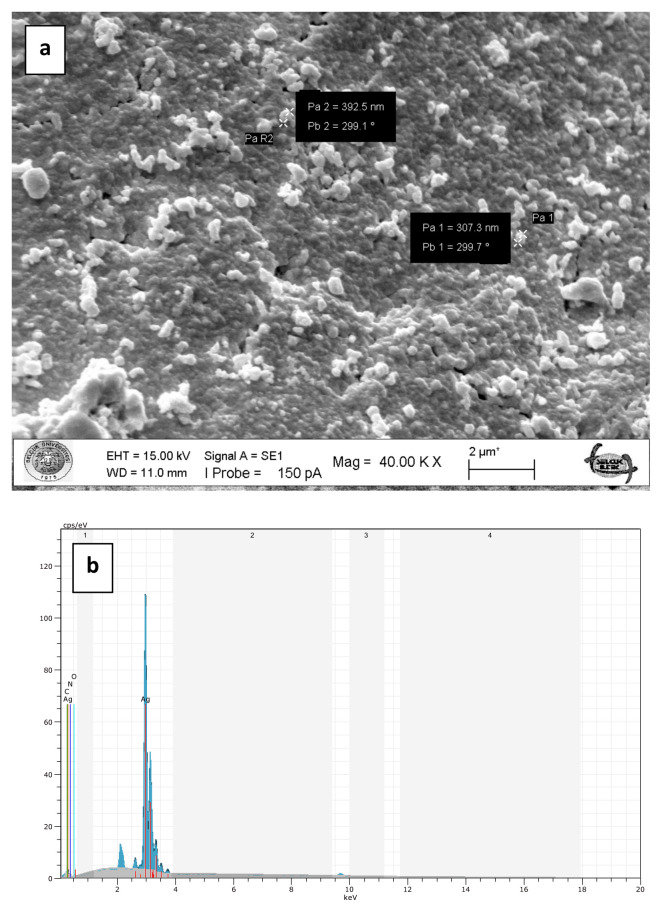
SEM image of the surface of Ag(0)-chitosan beads (a). SEM-EDX spectrum of Ag(0)-chitosan beads (b). The Ag peak is the indication of deposition of silver particles on the chitosan beads.

**Figure 7:**
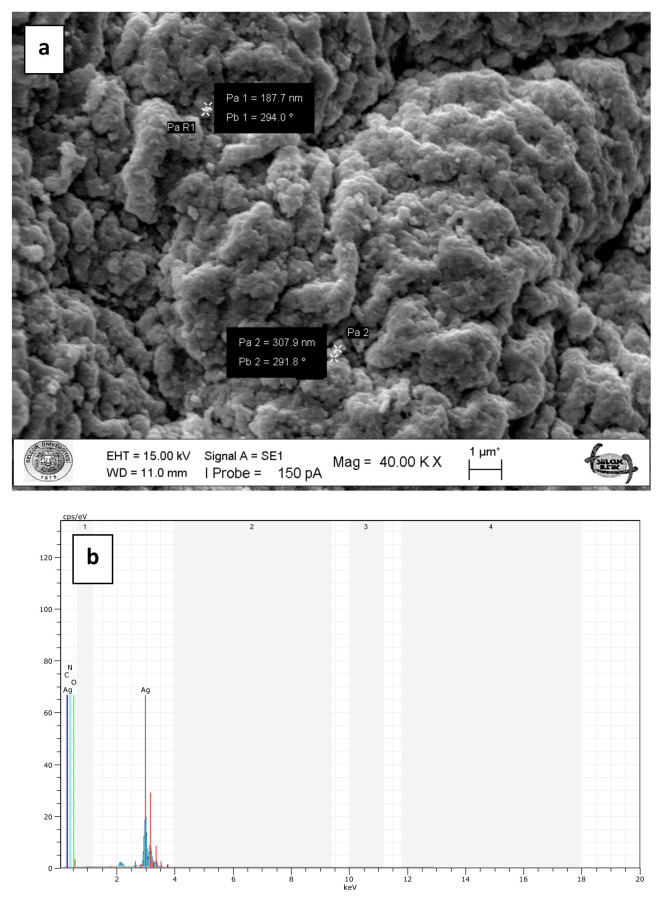
SEM image of the surface of Ag(0)-chitosan-*M. charantia* fruit powder beads (a). SEM-EDX spectrum of Ag(0)-chitosan-*M. charantia* fruit powder beads (b). The Ag peak is the indication of deposition of silver particles on the chitosan-*M. charantia* fruit powder beads.

**Figure 8:**
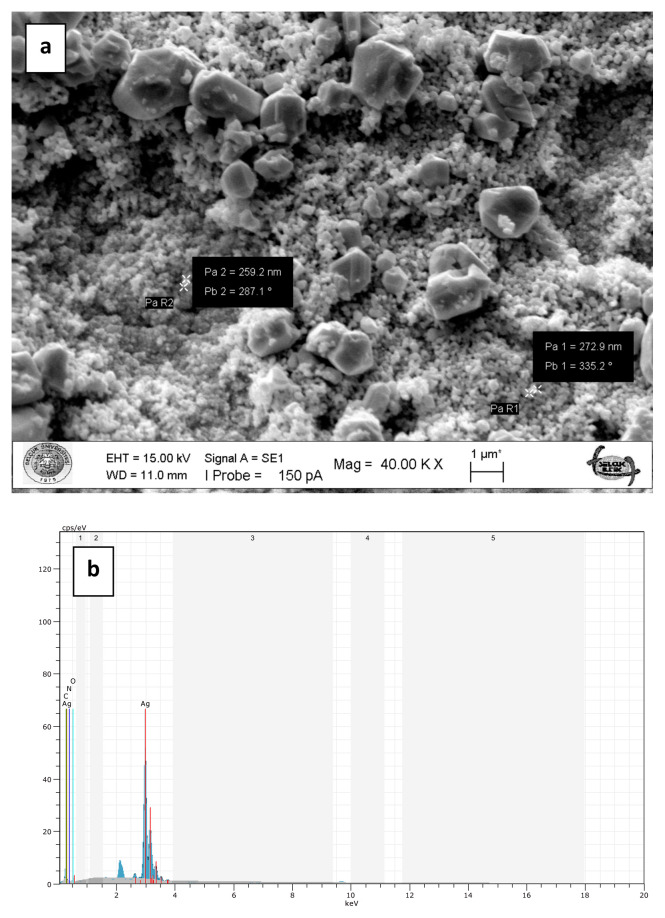
SEM image of the surface of Ag(0)-chitosan-carbon quantum dots beads (a). SEM-EDX spectrum of Ag(0)-chitosan-carbon quantum dots beads (b). The Ag peak is the indication of deposition of silver particles on the chitosan-carbon quantum dots beads.

**Figure 9:**
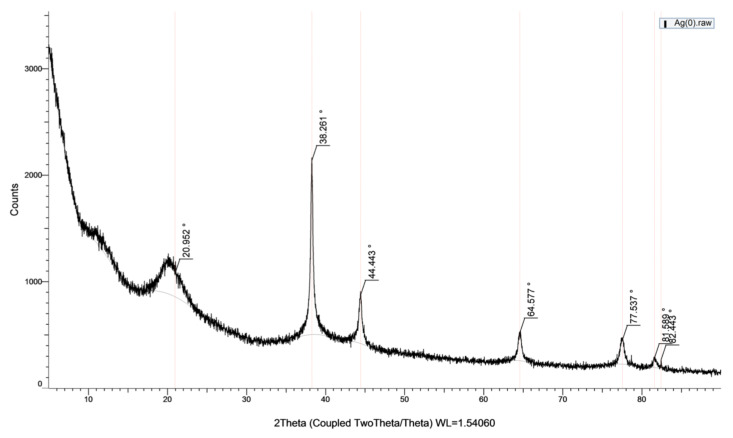
X-ray diffraction pattern of Ag(0)-chitosan beads.

To prepare chitosan-supported silver catalysts, glutaraldehyde cross-linked chitosan beads were loaded with silver ions in aqueous solution of silver nitrate and then silver ions were reduced onto the chitosan beads by means of sodium borohydride at 100°C. The metallic shiny appearance of the beads demonstrated that the reduction of the silver ions was successfully performed. Furthermore, the silver particles deposited on the surface of the beads are clearly seen in the SEM images (Figure 5 a–5f). The metallic silver particles on the surface of the beads gave them a much rougher appearance (Figures 5a and 5b). However, the high temperature during the reduction treatment led to deformations in the structure of the chitosan beads with *M. charantia* powder (Figure 5c and 5d). It is possible that microsized plant particles prevented homogenous and effective cross-linking of chitosan fibres. In case of Ag(0)-chitosan-CQDs beads, the nanosized CQDs were easily dispersed in the chitosan matrix by forming a more homogeneous mixture (Figures 5e and 5f). Therefore, the deformations in the structure of the plain chitosan beads and the chitosan beads with CQDs occurred to a lesser extent.SEM-EDX spectra of the plain chitosan beads (without *M. charantia* powder or CQDs) (Figures 6a and 6b), the chitosan beads with *M. charantia* powder (Figures 7a and 7b) and the chitosan beads with CQDs from *M. charantia* powder (Figures 8a and 8b) were obtained. The Ag peak in the EDX spectra is the indication of the deposition of silver particles on the chitosan beads. SEM images in Figures 6 and 8 show the surfaces of the catalysts. Ag(0)-chitosan beads had relatively smooth surfaces, whereas Ag(0)-chitosan-*M. charantia* fruit powder beads and Ag(0)-chitosan-carbon quantum dots beads had much rougher surfaces due to their particulate contents (i.e. *M. charantia* fruit powder or carbon quantum dots aggregates). All the catalysts were covered with randomly dispersed metallic silver particles most of which measures around 300 nm. However, there were some larger aggregates or clusters of silver on the surface of all catalysts. Additionally, X-ray diffraction (XRD) pattern of one of silver catalysts (Ag(0)-chitosan beads) is presented in Figure 9 to confirm the reduction of silver ions into its metallic form. The diffraction peaks at 38.26°, 44.44°, 64.58°, and 77.54° are ascribed to Ag(0), demonstrating the presence of the metallic silver particles on the catalyst. This observation confirms that silver ions deposited on the catalyst supports as metallic silver particles [30].

### 3.4. Reduction of nitroaromatic compounds over chitosan bead-supported silver catalysts

Reduction of nitroaromatic compounds to the corresponding amino compounds is thermodynamically favourable in the presence of hydrogen donor NaBH_4_. However, the kinetic barrier resulting from large potential gap between donor and acceptor molecules lowers the feasibility of this reaction. The kinetic barrier is overcome by addition of metallic particles. The presence of metallic particles catalyses the reduction of nitroaromatic molecules by facilitating electron relay from the donor borohydride to the acceptor nitro molecule [31]. In case of a phenolic compound like p-nitrophenol, the conversion of p-nitrophenol to p-aminophenol occurs via an intermediate 4-nitrophenolate ion formation.

**Figure 10:**
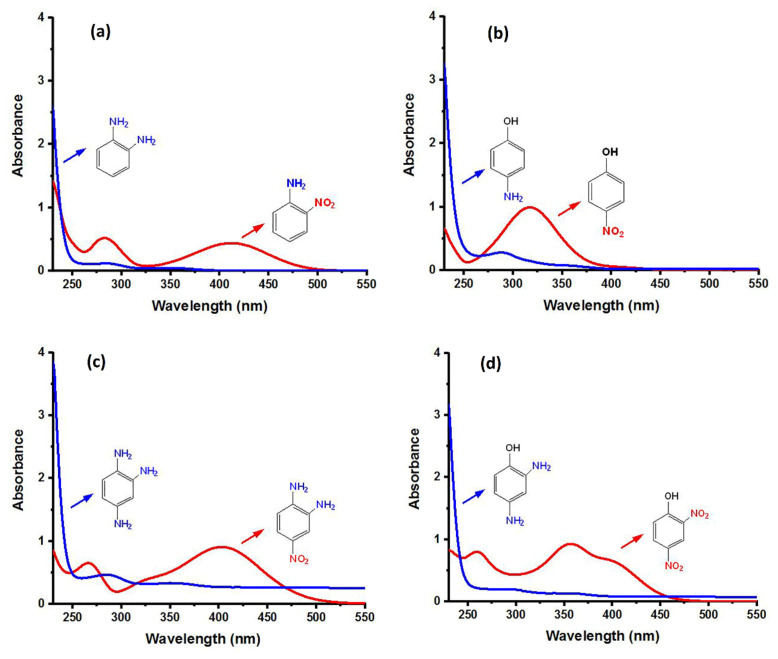
UV–vis spectra of 2-nitroaniline (a), 4-nitrophenol (b), 1,2-diamino-4-nitrobenzene (c) and 2,4-dinitrophenol (d) solutions, and their corresponding reduction products.

**Scheme 1:**
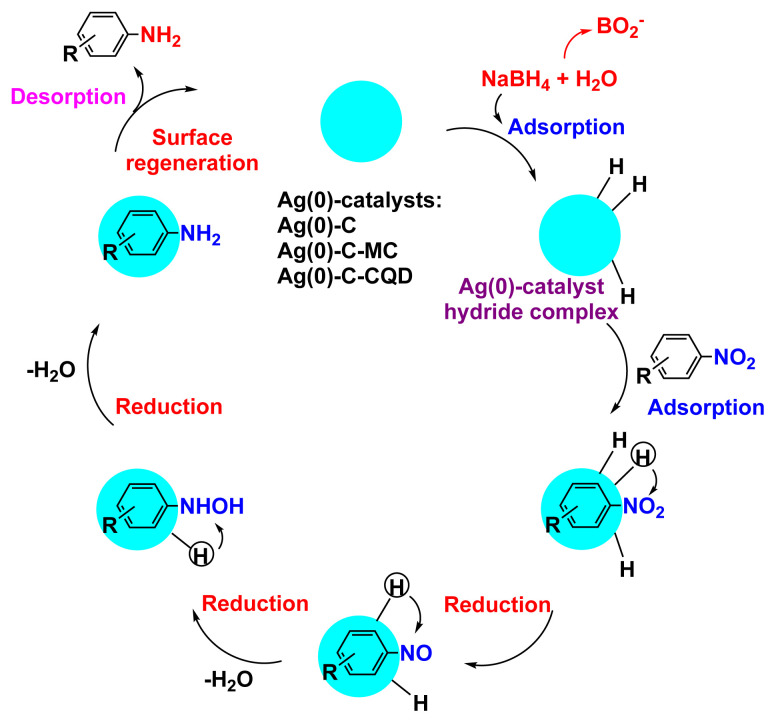
Possible reaction mechanism for the reduction of nitroaromatic compounds over Ag(0)-catalysts; C: chitosan, MC: *M. charantia* fruit powder, CQD: carbon quantum dots from *M. charantia* fruit powder.

The activity of the heterogeneous silver catalysts was tested in the reduction of toxic and recalcitrant nitroaromatic pollutants (4-nitrophenol, 2-nitroaniline, 1,2-diamino-4-nitrobenzene and 2,4-dinitrophenol) to the corresponding amines in aqueous media by using sodium borohydride as the hydrogen donor (Figures 10a–10d). The conversion time (referring to the end of the UV-vis measurement) for each reduction reaction and the reaction conditions are listed in Table 2. The catalytic activity of the silver catalysts with the plant particles or CQDs was higher than that of Ag(0)-chitosan beads. The catalytic activity of the silver catalysts increased with the amount of the loaded particles (*M. charantia* fruit powder or the CQDs from *M. charantia*), but the catalytic activity did not increase proportionally. It appeared that the catalytic activity occurred on the external catalyst layers. In reduction of 2-nitroaniline and 4-nitrophenol, Ag(0)-CQDs-chitosan beads showed a better performance. In case of 1,2-diamino-4-nitrobenzene and 2,4-dinitrophenol, shorter conversion times were recorded when Ag(0)-chitosan-*M. charantia* fruit powder was used. As given in Table 2, the catalysis results also confirmed the activity of Ag(0)-chitosan beads catalyst (without *M. charantia* powder or CQDs) for reduction of nitroaromatics to the corresponding amino compounds. However, the incorporation of microsized plant powder or nanoparticles (CQDs) greatly contributed to the reduction of nitroaromatics over Ag(0)-chitosan system. A possible reaction mechanism for the reduction of nitroaromatics over Ag(0)-catalysts is presented in Scheme 1.

In a previous study, magnetically separable chitosan microcapsules with silver nanoparticles were prepared for catalytic reduction of 4-nitrophenol [32]. The catalytic activity of the Fe_3_O_4_/CS–Ag catalyst showed a conversion efficiency as high as 98% within 15 min for the reduction of p-nitrophenol to p-aminophenol. In another study, the reduction of 4-nitrophenol in aqueous solution over 1 mg of Fe_3_O_4_@Chitosan–Ag nanocomposite system occurred in 20 min in the presence of sodium borohydride [33]. Alshehri et al. prepared Ag@MWCNTs-chitosan composite as heterogeneous catalyst for catalytic reduction of reduction of 4-nitrophenol [20]. The authors reported that 4-nitrophenol was converted into 4-aminophenol in 5 min in the presence of NaBH_4_. Vanaamudan et al. studied the catalytic performance of the chitosan-guar gum-silver nanocomposite for the reduction of p-nitrophenol in the presence of NaBH_4_ [34]. The reduction of p-nitrophenol to p-aminophenol was completed within 3 min and the catalyst showed catalytic activity to 3 cycles.

#### 3.4.1. Reusability of the silver catalysts

**Figure 11:**
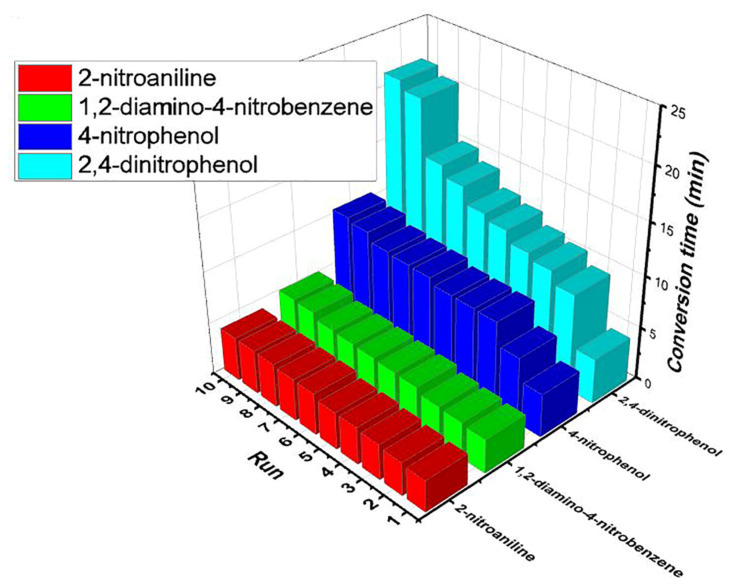
Reusability of the chitosan-based Ag(0) catalysts; Ag(0)-C-CQD-120 and Ag(0)-C-MC-120. Reduction of 2-nitroaniline and 4-nitrophenol was over Ag(0)-C-CQD-120 and 1,2-diamino-4-nitrobenzene and 2,4-dinitrophenol was over Ag(0)-C-MC-120.

Compared to other catalysts, the catalyst Ag(0)-C-CQD-120 showed higher activity in reduction of 2-nitroaniline to 1,2-diaminobenzene and 4-nitrophenol to 4-aminophenol (Table 2). In case of 1,2-diamino-4-nitrobenzene and 2,4-dinitrophenol, the activity of Ag(0)-C-MC-120 was recorded higher. The reusability of the catalysts was tested in the reactions where the catalyst was more efficient. In the reusability tests the same catalyst was used in ten subsequent runs. Following each run, the used catalyst was easily recovered by filtration and recycled ten times without much loss in its activity. Figure 11 presents the conversion time recorded for each run.

## 4. Conclusion

The study found that heterogeneous chitosan-based Ag(0) could be more efficient in reduction of nitroaromatic compounds when fine plant particles (from *M. charantia*) or nanomaterials like CQDs derived from *M. charantia* plant are incorporated into the chitosan gel matrix. The study also revealed that chitosan is an excellent matrix for incorporation of microsized or nanosized particles, indicating that chitosan is a convenient support biopolymer derivative for heterogeneous catalysis systems. Additionally, it was demonstrated that *M. charantia* plant particles could also serve as carbon source for fabrication of fluorescent carbon dots. In further studies Ag(0)-chitosan-CQDs systems can be tested in organic synthesis including carbon-carbon coupling reactions (Suzuki–Miyaura and Heck reactions), oxidations, and hydrogenations or degradation of organic pollutants.

## Figures and Tables

**Table 1:** The list of the silver catalysts.

Catalyst description	Catalyst contents			
	Chitosan (30 mL, 2% w/w)	*M. charantia* powder	CQDs from *M. charantia*	Metallic silver particles
Ag(0)-C	+	-	-	+
Ag(0)-C-MC-15	+	15 mg	-	+
Ag(0)-C-MC-30	+	30 mg	-	+
Ag(0)-C-MC-60	+	60 mg	-	+
Ag(0)-C-MC-120	+	120 mg	-	+
Ag(0)-C-CQD-15	+	-	15 mg	+
Ag(0)-C-CQD-30	+	-	30 mg	+
Ag(0)-C-CQD-60	+	-	60 mg	+
Ag(0)-C-CQD-120	+	-	120 mg	+

C: chitosan, MC: *Momordica charantia*

**Table 2:** Silver catalysed reduction of aromatic nitro compounds to their corresponding amino compounds over the plain Ag(0)-chitosan beads, Ag(0)-*M. charantia* powder-chitosan beads, and Ag(0)-CQDs-chitosan beads in the presence of hydrogen source (NaBH_4_) (*Conversion time* refers to the end of the UV-vis measurement for each reaction in minutes and seconds; the digits before the decimal separator denote minutes, and those after the decimal separator are seconds).

Reduction of 2-nitroaniline to 1,2-diaminobenzene					
Silver catalyst	Substrate*	Product	Catalyst mass (mg)	NaBH_4_ mass (mg)	Conversion time (minute.second)
Ag(0)-C	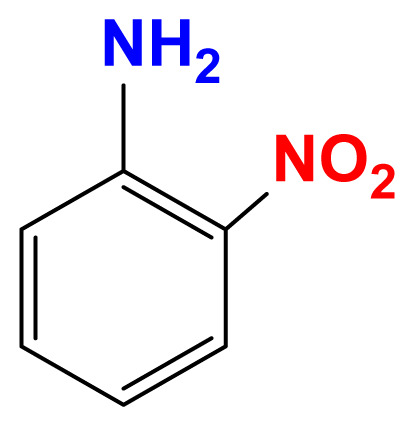	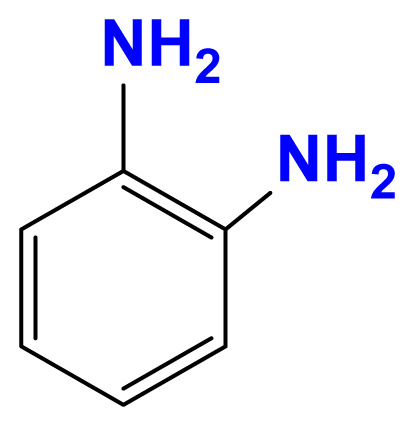	20.4	10.3	4.15
Ag(0)-C-MC-15			20.3	10.3	2.50
Ag(0)-C-MC-30			20.4	10.1	2.44
Ag(0)-C-MC-60			20.4	10.0	2.20
Ag(0)-C-MC-120			20.1	10.1	2.47
Ag(0)-C-CQD-15			20.2	10.4	2.40
Ag(0)-C-CQD-30			20.4	10.4	2.36
Ag(0)-C-CQD-60			20.4	10.4	2.19
Ag(0)-C-CQD-120			20.3	10.1	2.18
**Reduction of 4-nitrophenol to 4-aminophenol**					
Ag(0)-C	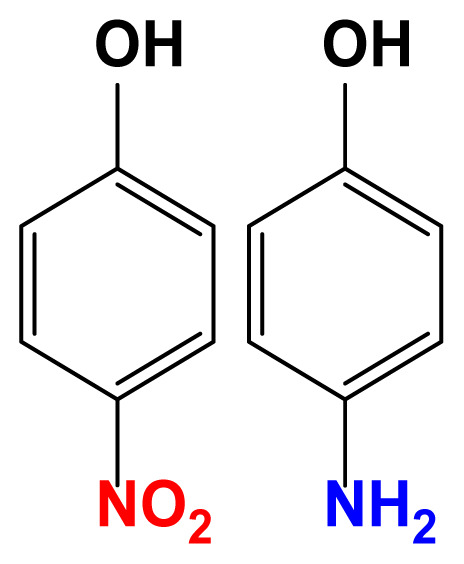		20.1	10.1	5.30
Ag(0)-C-MC-15			20.7	10.2	5.35
Ag(0)-C-MC-30			20.0	10.5	5.09
Ag(0)-C-MC-60			20.1	10.3	5.03
Ag(0)-C-MC-120			20.5	10.2	4.58
Ag(0)-C-CQD-15			20.6	10.1	5.00
Ag(0)-C-CQD-30			20.2	10.6	4.32
Ag(0)-C-CQD-60			20.3	10.1	4.10
Ag(0)-C-CQD-120			20.4	10.6	3.59
**Reduction of 1,2-diamino-4-nitrobenzene to 1,2,4-triaminobenzene**					
Ag(0)-C	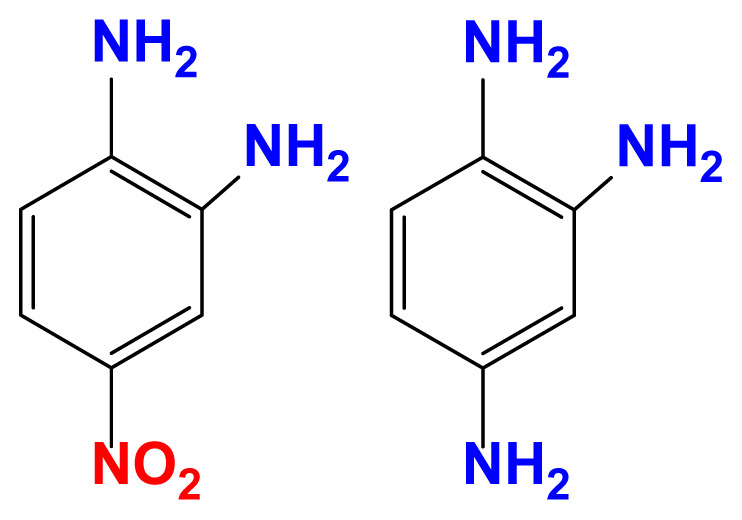		20.2	10.1	4.55
Ag(0)-C-MC-15			20.0	10.1	4.02
Ag(0)-C-MC-30			20.2	10.5	3.33
Ag(0)-C-MC-60			20.3	10.1	3.26
Ag(0)-C-MC-120			20.4	10.0	3.23
Ag(0)-C-CQD-15			20.3	10.3	3.55
Ag(0)-C-CQD-30			20.2	10.5	3.47
Ag(0)-C-CQD-60			20.2	10.6	3.49
Ag(0)-C-CQD-120			20.1	10.2	3.44
**Reduction of 2,4-dinitrophenol to 2,4-diaminophenol**					
Ag(0)-C	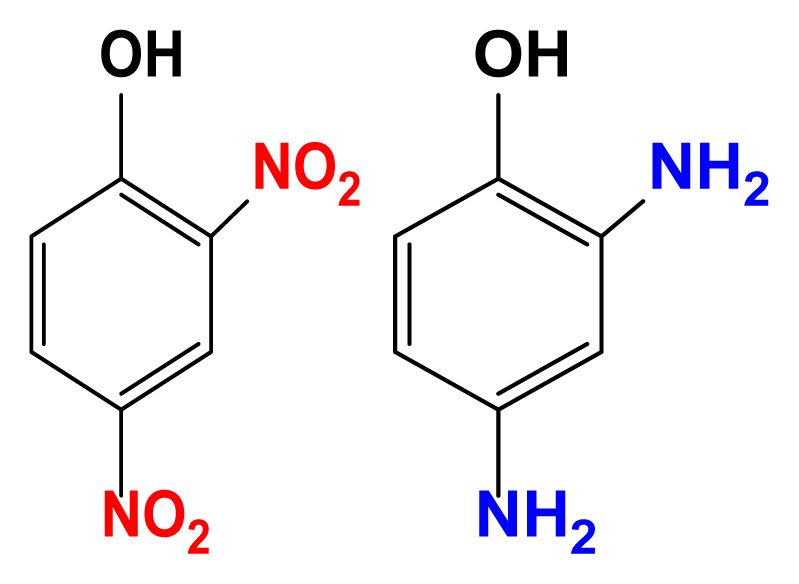		20.2	10.2	10.24
Ag(0)-C-MC-15			20.1	10.2	9.55
Ag(0)-C-MC-30			20.4	10.3	9.53
Ag(0)-C-MC-60			20.2	10.5	8.30
Ag(0)-C-MC-120			20.2	10.3	7.28
Ag(0)-C-CQD-15			20.2	10.3	9.12
Ag(0)-C-CQD-30			20.1	10.1	8.44
Ag(0)-C-CQD-60			20.1	10.2	8.36
Ag(0)-C-CQD-120			20.1	10.4	8.28

* 1.0 mL of aqueous solution of nitroaromatic compound (1.0 × 10^−4^ M, at 22 °C).
